# Exploring the potential of natural products in treating alopecia areata

**DOI:** 10.1016/j.jpha.2025.101539

**Published:** 2026-01-06

**Authors:** Baoli He, Yujia Weng, Peihua Luo, Zhifei Xu, Hao Yan, Bo Yang, Qiaojun He, Jiabin Lu, Xiaochun Yang

**Affiliations:** a Center for Drug Safety Evaluation and Research, College of Pharmaceutical Sciences, Zhejiang University, Hangzhou, 310058, China; b Nanhu Brain-computer Interface Institute, Hangzhou, 311100, China; c Innovation Institute for Artificial Intelligence in Medicine of Zhejiang University, College of Pharmaceutical Sciences, Zhejiang University, Hangzhou, 310058, China; d School of Medicine, Hangzhou City University, Hangzhou, 310015, China; e Hangzhou Institute of Innovative Medicine, College of Pharmaceutical Sciences, Zhejiang University, Hangzhou, 310058, China

**Keywords:** Natural products, Alopecia areata, Hair follicle immune privilege, Phytochemicals

## Abstract

Alopecia areata (AA) is an autoimmune disorder characterized by sudden hair loss, affecting millions worldwide. Conventional synthetic drug treatments are often limited by side effects, driving interest in natural alternatives. This review highlights terpenoids, flavonoids, and polyphenols as promising natural compounds that effectively promote hair regrowth in AA. These phytochemicals exert therapeutic effects primarily by activating pro-anagenic signaling pathways, notably the Wnt/β-catenin and phosphoinositide 3-kinase-protein kinase B (PI3K-Akt) pathways, while inhibiting catagen-associated pathways such as transforming growth factor-β (TGF-β), and modulating immune dysregulation through the suppression of Janus kinase-signal transducer and activator of transcription (JAK-STAT) signaling. This review has detailed the mechanisms through which these compounds support hair follicle regeneration, restore immune balance, and reduce inflammation. Collectively, the evidence highlights the significant therapeutic potential of natural products for AA, underscoring the need for further translational research.

## Highlights

• Systematically elucidates the pathogenic mechanisms of alopecia areata.
• Provides novel insights to guide future research on natural products for alopecia areata.
• Identifies promising molecular targets for the treatment of alopecia areata.

## Graphical abstract

**Figure:**
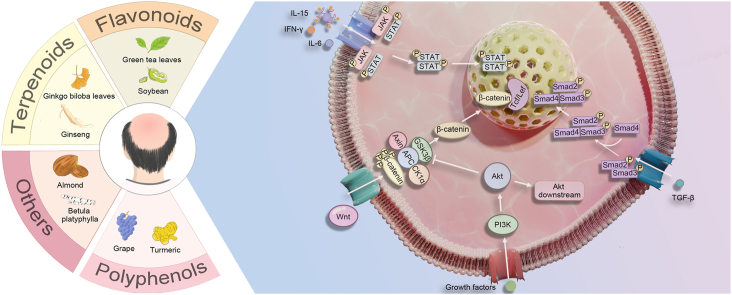

## 1 Introduction

Alopecia areata (AA) is a prevalent, acquired, and nonscarring type of hair loss that affects people of every generation and is intractable in severe or relapsing cases. Its global prevalence is estimated at approximately 0.1%–0.2% of the general population at any given time, with a lifetime risk of about 1.7% [1]. AA is associated with several diseases, such as hyperlipidemia, thyroid disease [2], inflammatory skin diseases [3], cancer [4], cardiovascular diseases [5], insulin resistance [6], ocular and auditory disorders [7], pregnancy and gynecological diseases [8].

Natural compounds exhibit multi-target pharmacodynamics and structural diversity, allowing them to simultaneously modulate interconnected pathological pathways in complex diseases such as AA. Unlike conventional single-target drugs, these compounds modulate key signaling pathways, including Janus kinase-signal transducer and activator of transcription (JAK-STAT) and Wnt, and also alter the cellular microenvironment to achieve broader therapeutic outcomes. These phytochemicals demonstrate distinct pharmacological advantages through their multi-target mechanisms and pathway modulations, exhibiting potent therapeutic effects across various autoimmune disorders [9,10]. Notably, natural product derivatives have shown significant promise in preclinical studies. For instance, amentoflavone induces ferroptosis in rheumatoid arthritis fibroblast-like synoviocytes (RA-FLS), thereby inhibiting their proliferation, migration, and invasion while reducing inflammation. This effect is mediated by the inhibition of peptidyl-prolyl cis/trans isomerase never in mitosis A (NIMA)-interacting 1 (PIN1), underscoring its potential as a candidate for rheumatoid arthritis (RA) treatment [11]. Some studies suggest that galangin holds potential as a therapeutic agent for treating RA [12]. A multitude of investigations showed that natural compounds represent promising strategies to fight against these autoimmune diseases [13]. Based on the above discussion, this review aims to highlight the natural compounds that play a significant role in regulating the mechanisms implicated in the development of AA, which is an autoimmune disease with a complex and multifactorial pathogenesis.

## 2 Pathogenesis of AA

**Fig. 1:**
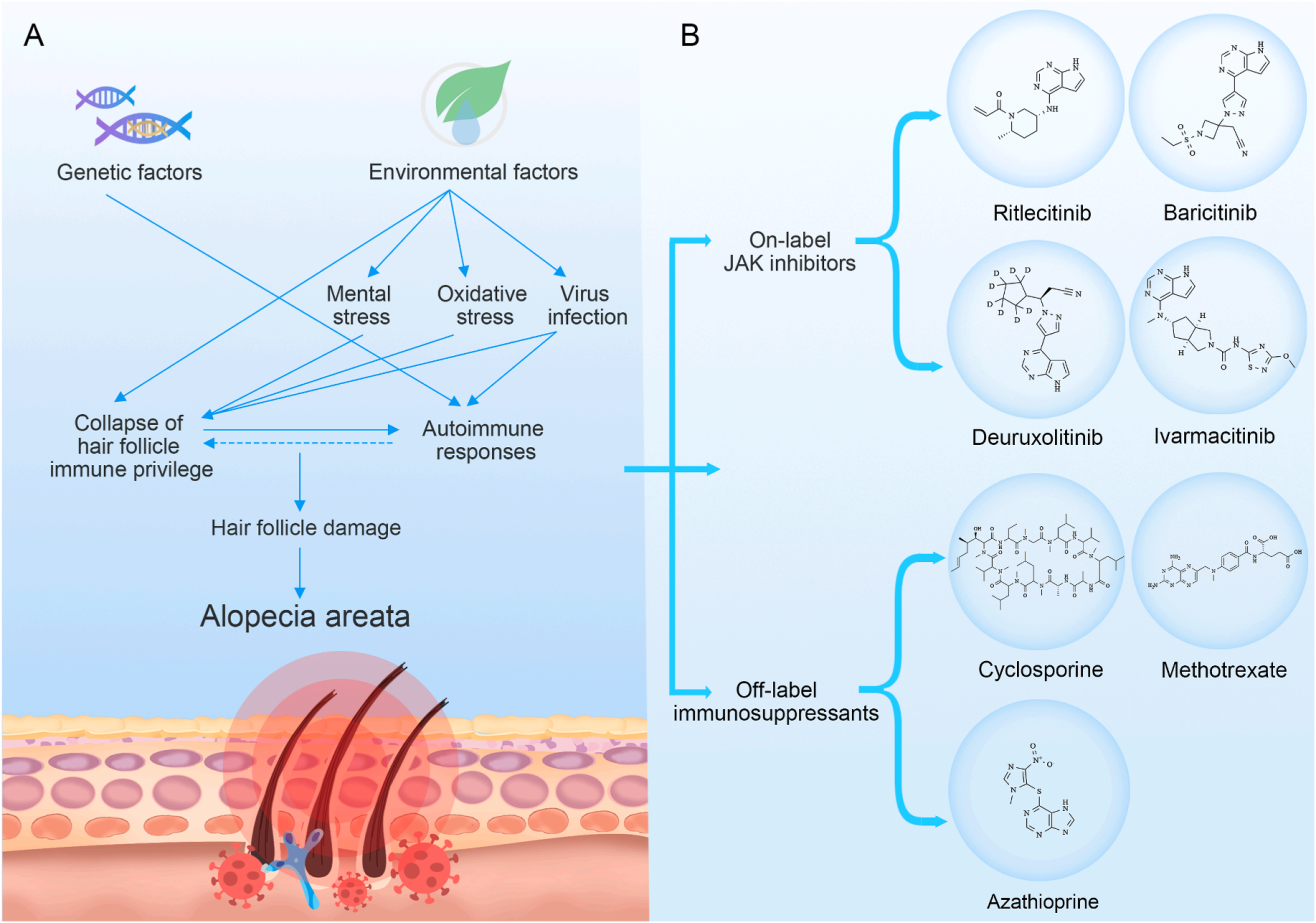
Pathogenesis and therapy framework for alopecia areata. (A) Network of disease biology: genetic predisposition and environmental factors converge on loss of hair follicle immune privilege (HFIP), permitting autoimmune responses, driving follicular injury, and culminating in alopecia areata. (B) Systemic pharmacotherapy mapped to this biology. On-label Janus kinase (JAK) inhibitors, including baricitinib, ritlecitinib, deuruxolitinib, and ivarmacitinib, with structures shown, block Janus kinase-signal transducer and activator of transcription (JAK-STAT) signaling and represent first-line systemic treatment for severe disease. Off-label immunosuppressants, including cyclosporine, methotrexate, and azathioprine, with structures shown, provide broad immunosuppression when JAK inhibitors are unsuitable. Short-course systemic glucocorticoids for induction or bridging and low-dose oral minoxidil as an adjunct are noted but not depicted.

AA is an immune-mediated disorder that targets hair follicles (HFs) and results in nonscarring hair loss [14]. Its pathogenesis involves a complex interplay of genetic predisposition, environmental triggers, and immune dysregulation, which collectively drive the collapse of hair follicle immune privilege (HFIP) and amplify interferon-γ (IFN-γ)/interleukin-15 (IL-15)-mediated JAK-STAT signaling. Mapped onto this biology, on-label systemic options now include oral JAK inhibitors (JAKi), such as baricitinib, ritlecitinib, deuruxolitinib, and ivarmacitinib, which antagonize the JAK-STAT axis and constitute first-line pharmacotherapy [[15], [16], [17]]. In cases where JAKi therapy is contraindicated, inaccessible, or not tolerated, off-label alternatives such as cyclosporine, methotrexate, and azathioprine are commonly used. Short-course systemic glucocorticoids may be employed for induction or bridging therapy, while low-dose oral minoxidil is generally considered adjunctive rather than disease-modifying [15] (Fig. 1). Although these treatments can induce hair regrowth, their clinical utility is often limited by variable patient response, relapse, and safety or tolerability concerns, underscoring the need for better-tolerated, mechanism-informed therapeutic strategies.

### 2.1 Genetic factors

Genetic factors play a crucial mechanistic role in the pathogenesis of AA. Familial aggregation and twin studies consistently demonstrate a strong heritable component, supporting the significant contribution of genetic predisposition to disease susceptibility [[18], [19], [20], [21]]. Genome-wide association studies have further revealed that AA is a polygenic autoimmune disorder, in which multiple loci collectively drive disease risk through immune dysregulation and loss of HFIP.

The most significant associations lie within the major histocompatibility complex (MHC) on chromosome 6p, particularly in human leukocyte antigen (HLA) class II genes such as *HLA-DRB1*, which are central to antigen presentation and immune regulation. Beyond the MHC, several non-HLA immune-related susceptibility loci have been identified, including cytotoxic T-lymphocyte-associated protein 4 (*CTLA4*), interleukin-2/interleukin-21 (*IL-2/IL-21*), and interleukin-2 receptor subunit α (*IL2RA*), which regulate T-cell activation and peripheral tolerance. Mutations or polymorphisms in these genes contribute to excessive T-cell activation, particularly cytotoxic CD8^+^ T cells, and impair regulatory T-cell function, thereby promoting autoimmunity [[22], [23], [24], [25]].

A distinctive hallmark is the chromosome-6q cluster of UL16-binding proteins (ULBPs) which includes ULBP3 and ULBP6, whose stress-inducible ligands engage natural killer group 2, member D (NKG2D) on CD8^+^ T cells and natural killer (NK) cells; aberrant follicular expression is thought to precipitate collapse of HFIP and permit direct cytotoxic attack. Additional signals related to follicular architecture and stress response, such as syntaxin-17 (STX17), peroxiredoxin-5 (PRDX5), and melanin-concentrating hormone receptor 2 (MCHR2), may modulate follicular susceptibility by influencing oxidative stress, autophagy, and pigment signaling pathways [25]. Together, these findings indicate that AA arises from a convergence of genetic pathways governing both innate and adaptive immunity, with downstream effects frequently mediated through JAK-STAT signaling, especially via IL-15-driven activation of CD8^+^ T cells. This mechanistic insight provides a strong biological rationale for the clinical efficacy of JAKi in restoring immune tolerance and promoting hair regrowth in AA.

### 2.2 Environmental factors

Various environmental triggers have been identified as potential catalysts for the onset or exacerbation of AA, such as viral infections, trace elements or micronutrients, immunization status, and mental stress, which are considered to play a role in influencing the disease [26]. Smoking elevates AA risk through immune-mediated inflammation, whereas alcohol consumption exhibits a dual role, potentially protecting against AA onset while exacerbating cutaneous inflammation. Sleep disturbances are associated with elevated risk via immune dysregulation, whereas obesity amplifies susceptibility through metabolic-inflammatory interplay. Dietary components exhibit divergent impacts, with omega-3 fatty acids demonstrating protective potential and gluten intake correlating with disease exacerbation [27]. Emerging evidence suggests that HF and gut microbiota, along with oxidative stress, contribute to AA pathogenesis. Oxidative stress, in particular, may initiate immune dysregulation, contributing to hair loss and disease progression [28,29].

### 2.3 Immunopathogenesis of AA

AA is increasingly recognized as an organ-specific autoimmune disease in which a breakdown of HFIP is now thought to play a central role and be a crucial precondition for the development of AA [30,31]. It results from a multifactorial interaction between genetic predisposition and environmental triggers that disrupt the follicular immune balance. The HF, a neuroectodermal-mesodermal-derived mini-organ [32], normally sustains an immune-privileged status from the bulge (stem cell niche) to the bulb. This immune privilege (IP) establishes a specialized microenvironment that restricts immune cell infiltration and suppresses autoreactive T cell activation through several mechanisms [31], including constitutive suppression of MHC class I expression in proximal outer root sheath and matrix cells, a critical protective feature lost in AA, where upregulated MHC molecules expose autoantigens to CD8^+^ T cells [33].

The local immunoinhibitory environment is further reinforced by the secretion of immunosuppressive molecules, known as “IP guardians”, including α-melanocyte-stimulating hormone (α-MSH), transforming growth factor-β1 (TGF-β1), indoleamine-2,3-dioxygenase (IDO), protein red encoded by IK gene (red/IK), IL-10, IL-15, calcitonin gene-related peptide (CGRP), insulin-like growth factor-1 (IGF-1), and somatostatin [34,35].

**Fig. 2:**
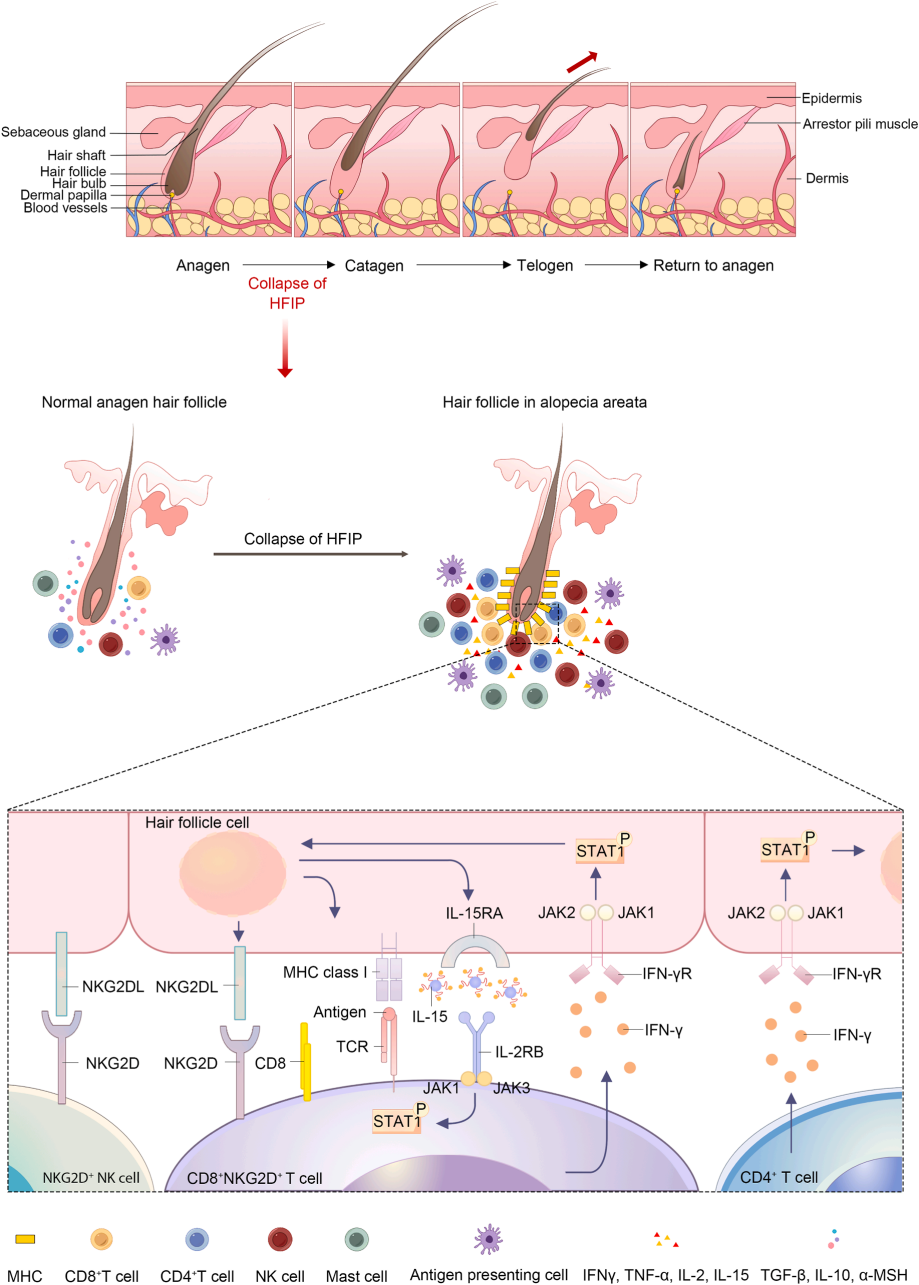
Collapse of hair follicle immune privilege. In healthy follicles, immune privilege is robust in anagen and wanes in catagen and telogen. Breakdown is characterized by increased major histocompatibility complex (MHC) class I and II and induction of natural killer group 2, member D ligands (NKG2DL) together with loss of local immunoinhibitory signals such as α-melanocyte-stimulating hormone (α-MSH), and transforming growth factor-β (TGF-β). These changes expose follicular antigens and permit infiltration by CD8^+^ and CD4^+^ T cells, antigen-presenting cells and mast cells. The ensuing interferon-γ (IFN-γ) and interleukin-15 (IL-15) signaling amplifies Janus kinase-signal transducer and activator of transcription (JAK-STAT) activity, disrupts hair-cycle control, accelerates anagen termination and culminates in structural injury of the follicle. HFIP: hair follicle immune privilege; NKG2D: natural killer group 2, member D; TCR: T-cell receptor; STAT1: signal transducer and activator of transcription 1; IL-2: interleukin-2; IL-15: interleukin-15; IL-10: interleukin-10; IL-2RB: IL-2 receptor subunit-β; IL-15RA: IL-15 receptor subunit-α; JAK1: Janus kinase 1; JAK3: Janus kinase 3; IFN-γR: interferon-γ receptor; TNF-α: tumor necrosis factor-α.

HFIP is maintained during anagen but is lost during the telogen and catagen phases of the hair cycle (Fig. 2). All mature follicles undergo a growth cycle that consists of phases [36]. The anagen phase involves remodeling of the lower follicle and production of a new hair fiber, determining the hair length on the scalp. This is followed by a short transitional catagen phase, during which hair follicles shrink and detach, and then by telogen, a resting phase [[36], [37], [38]].

HFIP collapse occurs when signaling pathways that maintain this IP are overwhelmed by pro-collapse signals, such as the proinflammatory cytokine IFN-γ, which triggers HFIP breakdown by enhancing the expression of MHC I and II [39].

HFIP collapses, causing inflammatory cells to swarm and attack the hair bulb in what is known as the “swarm of bees”, which is thought to be essential for the onset of AA [40]. In AA, HF cells and dendritic cells exhibit increased MHC class I and II activity, presenting autoantigens to CD8^+^ T cells, CD4^+^ T cells, and NK cells [41].

In AA, the collapse of IP in HFs is a key event in disease development. This process is driven by the upregulation of MHC I/II, adhesion molecules, and NKG2DL, as well as increased secretion of pro-inflammatory cytokines like IFN-γ, IL-15, tumor necrosis factor-α (TNF-α) and IL-2. Simultaneously, the mechanisms that maintain IP are weakened. The enhanced presentation of autoantigens by melanin-producing anagen HF cells further contributes to the loss of IP. Following this collapse, HF cells release cytokines and chemokines that recruit CD4^+^ T cells and NK cells, thereby amplifying inflammation and immune-mediated damage [42]. This cellular infiltrate intensifies local inflammation through IFN-γ-driven upregulation of MHC expression and IL-15-mediated activation of the JAK-STAT pathway in CD8^+^ T cells. The resulting positive feedback loop stimulates cytotoxic granzyme B release, clonal T cell expansion, and ultimately, follicular destruction and the clinical manifestations of AA [43].

Conceptually, AA emerges when pro-destructive cues outweigh the follicle's immune-privilege safeguards. Upstream interferon signals elevate MHC class I/II and NKG2DL such as major histocompatibility complex class I polypeptide-related sequence A (MICA), while adhesion molecules and mast-cell mediators further lower the activation threshold for cytotoxic lymphocytes. In parallel, the local “IP guardians” are diminished. Once this balance tips, anagen follicles become immunogenic: enhanced antigen displays and IFN-inducible C-X-C chemokine receptor 3 (CXCR3) ligands recruit and license CD8^+^ T cells and NK cells, IL-15 sustains JAK-STAT activation, and a self-amplifying loop of granzyme-mediated cytotoxicity and clonal expansion culminates in peribulbar damage [31]. The temporal progression from subclinical IP dysregulation to overt AA manifestation likely depends on the threshold accumulation of these immunopathological alterations, though precise sequence determinants remain to be fully elucidated.

## 3 Natural products with therapeutic potential for AA

### 3.1 Terpenoids

Terpenoids constitute a vast category of secondary plant metabolites that exhibit considerable structural diversity. Derived from isoprene units (C-5), these compounds possess a broad spectrum of biological properties, such as antioxidant, antimicrobial, anti-inflammatory, anti-allergic, anticancer, antimetastatic, and antiangiogenic activities, and the ability to induce apoptosis [44]. With its unique molecular structure and extensive medicinal value, terpenoids are gradually becoming a new hope for AA treatment (Table 1) [[45], [46], [47], [48], [49], [50], [51], [52], [53], [54], [55], [56], [57], [58], [59], [60], [61]]. Despite their diverse structures, terpenoids demonstrate a unified mechanism of action against AA, synergistically promoting HF function and countering inflammatory drivers. Their efficacy is largely rooted in activating anagen-promoting pathways, notably Wnt/β-catenin and phosphoinositide 3-kinase-protein kinase B (PI3K-Akt), to enhance dermal papilla cell (DPC) proliferation and initiate the telogen-to-anagen transition. Concurrently, they ameliorate the autoimmune component by inhibiting pivotal signaling cascades such as JAK-STAT and nuclear factor-kappa B (NF-κB), which reduces perifollicular inflammation and immune-mediated damage. This dual approach of fostering follicular regeneration while quelling the inflammatory response underscores their potential as therapeutics.

**Table 1:** Terpenoids with therapeutic potential for alopecia areata.

Compounds	Subclass	Sources	Mechanisms	Observed efficacy	Stage of evidence	Experimental models	Chemical construction	Refs.
Cedrol	Sesquiterpene	Ginger	• Reduce JAK3/STAT3 expression • Activate the Wnt3a/β-catenin germinal center	Promote hair regrowth and follicular recovery in AA mouse model	Preclinical (*in vivo*, *in vitro*)	C57BL/6 mice, HaCaT cells		[[45], [46], [47]]
Ginsenoside Rb1	Triterpenoid saponins	Ginseng	• Suppress BMP4 expression in DPCs • Stimulate the transition from the telogen phase to anagen phase of the hair cycle	Promote DPC and ORSC proliferation and induce anagen transition	Preclinical (*in vitro*)	hDPCs, hORSCs		[48]
Ginsenoside Rg1								
Ginsenoside Re								
Ginsenoside Rg4	Triterpenoid saponins	Ginseng	• Activate the Akt/GSK3β/β-catenin signaling pathway • Increase cell viability and spheroid size, and expression of hair growth genes	Enhance DPC viability, spheroid size	Preclinical (*in vitro*)	3D human DP spheroid model		[49]
Ginsenoside CK	Triterpenoid saponins	Ginseng	• Inhibit p53-mediated apoptosis • Activate the Wnt/β-catenin pathway	Increase DPC viability and hair growth gene expression	Preclinical (*in vivo*, *in vitro*)	C57BL/6 mice, HaCaT cells		[50]
*Panax notoginseng saponins*	Triterpenoid saponins	*Panax notoginseng*	• Activate the Wnt/β-catenin pathway	Significant anagen induction and follicular growth in mice	Preclinical (*in vivo*)	C57BL/6 mice	N/A	[51]
Bilobalide	Sesquiterpene trilactone	*Ginkgo biloba*	• Increase Akt and β-catenin activities	Enhance hair follicle elongation, DP cell viability, and VEGF secretion	Preclinical (*ex vivo*, *in vitro*)	Mink HFs, DP cells		[[52], [53]]
Ginkgolide B	Diterpene lactone	*Ginkgo biloba*	• Activate Akt, ERK1/2, and β-catenin signaling	Stimulate HF elongation and DP cell proliferation in mink HF model	Preclinical (*ex vivo*, *in vitro*)	Mink HFs and DP cells		[53]
Oleanolic acid	Pentacyclic triterpenoid	*Olea europaea* L.	• Stimulate follicle cell proliferation by increasing the Wnt/β-catenin pathway	Promote follicular elongation and proliferation in culture	Preclinical (*ex vivo*)	Human HF organ culture model		[54]
Costunolide	Sesquiterpene lactone	Aucklandiae Radix	• Inhibit 5α-reductase activity • Increase β-catenin and Gli1 expression • Suppress TGF-β1-induced Smad-1/5 phosphorylation	promote follicular regrowth in cell and animal models	Preclinical (*in vivo, in vitro*)	C57BL/6 mice, HFDPCs		[55]
Enmein	Diterpenoid	*Isodon japonicus Hara*	• Activate p-Akt, p-GSK-3β, β-catenin, driving the proliferation of HFDPCs via the Akt/GSK-3β/β-catenin transduction pathway • Enhance VEGF expression	Enhance proliferation and survival of DP cells	Preclinical (*in vitro*)	HFDPCs		[56]
Loliolide	Monoterpenoid	Carotenoids	• Induce hair growth in DP cells through the Akt/β-catenin pathway	Promote DP spheroid growth and follicular activation	Preclinical (*in vitro*)	3D DP spheroid model		[57]
Lycopene	Tetraterpenoid	*Tomatoes, watermelon*, etc.	• Increase VEGF, keratinocyte growth factor, and IGF-1	Induce early anagen and comparable effect to minoxidil in mice	Preclinical (*in vivo*)	C57BL/6 mice		[58]
Cucurbitacins	Triterpenoid	Cucurbitaceae plants	• Inhibit Fgf18 expression	Accelerate telogen–anagen transition in murine model	Preclinical (*in vivo*)	C57BL/6 mice		[59,60]
Araliadiol	Pentacyclic triterpenoid	Ivy	• Promote cell proliferation and hair growth • Increase the expression of related proteins and hair-growth-promoting factors • Depend on activation of the p38/PPAR-γ signaling pathway	Promote HF cell proliferation and growth *in vitro*	Preclinical (*in vitro*)	Human HF stem cells, hDPCs		[61]

AA: alopecia areata; JAK3: Janus kinase 3; STAT3: signal transducer and activator of transcription 3; Wnt: wingless-related integration site; Wnt3a: wingless-related integration site 3a; BMP4: bone morphogenetic protein 4; DPCs: dermal papilla cells; DPC: dermal papilla cell; hDPCs: human dermal papilla cells; hORSCs: human outer root sheath cells; Akt: protein kinase B; p-Akt: phosphorylated Akt; GSK3β: glycogen synthase kinase 3 beta; DP: dermal papilla; p-GSK-3β: phosphorylated GSK-3β; p53: tumor protein p53; N/A: not applicable; β-catenin: beta-catenin; VEGF: vascular endothelial growth factor; HFs: hair follicles; HF: hair follicle; ERK1/2: extracellular signal-regulated kinase 1/2; Gli: glioma-associated oncogene homolog; Gli1: glioma-associated oncogene homolog 1; TGF-β1: transforming growth factor beta 1; Smad-1/5: mothers against decapentaplegic homolog 1/5; HFDPCs: hair follicle dermal papilla cells; IGF-1: insulin-like growth factor 1; FGF18: fibroblast growth factor 18; PPAR-γ: peroxisome proliferator-activated receptor gamma.

#### 3.1.1 Cedrol (CE)

CE is a small sesquiterpene molecule in ginger that exhibits health benefits, including anti-inflammatory, anti-arthritic, and pain-relieving properties. Ginger itself has a traditional history in East Asia for combating hair loss and stimulating hair growth [45], potentially linked to its ability to improve the oxidant/antioxidant balance in erythrocytes and lymphocytes, as well as trace element status, observed in AA patients [46].

A comparative study on the efficacy of oral administration versus topical application of CE in C57BL/6 mice with cyclophosphamide-induced AA demonstrated that oral CE administration significantly enhanced hair regrowth, mitigated follicular damage, and accelerated cellular proliferation, outperforming topical CE application. At the molecular level, oral CE administration downregulated the JAK3-STAT3 signaling pathway while simultaneously activating the Wnt3a/β-catenin signaling cascade in HF cells, thereby promoting follicular cell proliferation and effectively reversing AA progression [47], which could improve the oxidant/antioxidant balance of the erythrocytes and lymphocytes and trace elements status in patients with AA.

Collectively, the demonstration of efficacy in an AA model together with modulation of core inflammatory and pro-regenerative pathways positions CE as a highly AA-relevant natural therapeutic candidate.

#### 3.1.2 Ginsenosides

Ginsenosides, the principal bioactive terpenoids in *Panax ginseng* C. A. Mey*.*, underpin its nutraceutical and pharmaceutical significance [62]. Substantial preclinical evidence supports the role of ginsenosides in HF biology, primarily through modulation of DPC dynamics and apoptotic regulation [[63], [64], [65], [66], [67]].

Extracts rich in ginsenosides demonstrate potent hair growth effects. Red ginseng extract (RGE) contains a wide variety of ginsenosides, which promote hair growth by stimulating dermal papilla cells (DPCs) proliferation and enhancing skin functions. RGE treatment significantly upregulated the expression of β-catenin, p-GSK3β, cyclin D1 (CCND1), cyclin E (CCNE1), B-cell lymphoma 2 (Bcl-2), phospho-extracellular signal-regulated kinase (p-ERK), and phospho-protein kinase B (p-Akt), which are associated with hair growth hair growth-promoting mechanisms of RGE through stimulating dermal papilla [68]. Similarly, red ginseng oil, extracted from red ginseng, has been demonstrated in C57BL/6 mice to effectively promote hair regeneration by inducing early telogen-to-anagen transition and significantly increasing the density and bulb diameter of HFs [69].

A study examined the impact of *P. ginseng* extract (PGE) and its ginsenosides Rb1, Rg1, and Re on human dermal papilla cells (hDPCs) and outer root sheath cells (ORSCs). PGE and the three ginsenosides stimulated the proliferation of DPCs and ORSCs and suppressed bone morphogenetic protein 4 (*BMP4*) mRNA expression in DPCs but did not affect fibroblast growth factor (*FGF18*) mRNA expression in ORSCs and noggin (*NOG*) mRNA expression in DPCs. Collectively, these effects likely contributed to stimulating the transition from telogen to anagen phase of the hair cycle [48]. Furthermore, in a three-dimensional (3D) DPC model, ginsenoside Rg4 enhances cell viability, size, and hair growth gene expression, and activates the PI3K-Akt pathway, which phosphorylates and inhibits GSK3β, thereby leading to Wnt/β-catenin signaling activation [49].

Complementing these findings, a focused study on ginsenoside CK revealed its efficacy against androgenetic alopecia via a dual mechanism: inhibiting p53-mediated apoptosis and activating the Wnt/β-catenin pathway, thereby promoting HF survival and regeneration [50].

Collectively, by targeting critical pathways in HF cycling (Wnt/β-catenin, PI3K-Akt), modulating DPC function, inhibiting apoptosis, promoting anagen entry, and demonstrating significant hair growth stimulation, ginsenosides present a compelling case as promising natural therapeutic agents for AA.

#### 3.1.3 *Panax notoginseng* saponins (PNS)

PNS represent the active constituents extracted from the traditional herb, *Panax notoginseng (Burkill) F.H.Chen ex C.H.Chow*. PNS are considered a potent compound for promoting hair growth, and its function has been confirmed *in vivo*. In a C57BL/6J mouse model, 8% PNS most effectively promoted hair follicle growth, accompanied by enhanced cellular metabolism and activation of Wnt/β-catenin signaling. Immunohistochemistry and immunofluorescence analyses showed increased cell proliferation and apoptosis in the 8% PNS-treated group. β-catenin, Wnt10b, and lymphoid enhancer-binding factor 1 (LEF1) were upregulated, while Wnt5a was suppressed, especially in the 8% group. From the above experiment, PNS may promote hair growth via Wnt/β-catenin [51].

Given its potent stimulation of HF growth through the crucial Wnt/β-catenin pathway, which is a key regulator often implicated in AA pathogenesis, PNS emerges as a highly promising natural candidate for developing novel AA therapeutics.

#### 3.1.4 Ginkgolide B (GKB) and bilobalide (BB)

GKB and BB are two functional components found in the leaves of *Ginkgo biloba*. GKB, a natural substance extracted from *Ginkgo biloba* L., is a dietary diterpene with multiple pharmacological activities. It possesses several beneficial effects such as anti-inflammatory, anti-allergic, antioxidant, and neuroprotective effects [70]. BB, a natural sesquiterpene trilactone derived from *Ginkgo biloba* leaves, exhibits relatively good water solubility and finds widespread application in both the food and pharmaceutical industries. Over the past decade, numerous studies have been conducted on its pharmacological properties, revealing a broad spectrum of activities including neuroprotective, antioxidant, anti-inflammatory, anti-ischemic, and cardioprotective effects [52].

In a study, mink HFs and isolated dermal papilla (DP) cells were treated with GKB or BB to evaluate their effects on hair growth. Both compounds stimulated HF elongation and enhanced DP cell viability. Additionally, they increased vascular endothelial growth factor (VEGF) secretion, suggesting a pro-angiogenic role. Mechanistically, GKB activated Akt, extracellular signal-regulated kinase 1/2 (ERK1/2), and β-catenin signaling, whereas BB selectively upregulated Akt and β-catenin. Despite their distinct mechanisms in modulating β-catenin signaling, both compounds promoted DP cell survival and accelerated the HF cycle [53]. The dual capacity of GKB and BB to enhance DP cell viability, activate pro-regenerative signaling pathways, and elevate VEGF levels highlights their potential as novel therapeutic agents for AA.

#### 3.1.5 Oleanolic acid (OA)

OA, a plant-derived pentacyclic triterpenoid, exhibits broad therapeutic potential, including benefits for dyslipidemia, diabetes, metabolic syndrome, and antimicrobial, anticancer, and anti-inflammatory activities [71]. Research shows that 1 or 10 μg/mL OA can make HFs' shafts elongate and enter the growth stage. It also increases the positive rate of Ki-67 and the expression of β-catenin. This suggests OA may promote hair growth by stimulating the follicle cell proliferation through the Wnt/β-catenin pathway [54]. These findings identify OA as a promising candidate for AA treatment, primarily by stimulating HF cell proliferation via activation of the Wnt/β-catenin signaling pathway.

#### 3.1.6 Costunolide (COS)

COS, a naturally occurring sesquiterpene lactone found in Aucklandiae Radix, has been the subject of extensive research due to its diverse biological activities. Various preclinical studies have shown that this compound exhibits antioxidative, anti-inflammatory, anti-allergic, and neuroprotective properties, and promotes hair growth [72]. It promotes hair growth by stimulating human hair follicle dermal papilla cells (HFDPCs) proliferation. COS inhibits 5α-reductase activity, increases β-catenin and glioma-associated oncogene homolog 1 (Gli1) expression, and suppresses TGF-β1-induced phosphorylation of mothers against decapentaplegic homolog 1 (Smad1) and homolog 5 (Smad5) in human HFDPCs. In reconstitution assays, COS induced HF formation and significantly improved hair growth in shaved C57BL/6 mice. This dual regulation of anagen-promoting (Wnt/Hedgehog) and catagen-inhibiting TGF-β pathways positions COS as a novel multi-target phytotherapeutic candidate for AA [55].

#### 3.1.7 Enmein

Enmein is a diterpenoid derived from the plant *Isodon japonicus Hara*, commonly used in dietary supplements and folk medicine. It has demonstrated antibacterial, anti-inflammatory, antitumor, and immunosuppressive effects [73]. Additionally, methanol extracts from Isodonis Herba significantly promote the proliferation of HFDPCs. Isolated compounds, including enmein, isodocarpin, nodosin, and oridonin, exhibited similar proliferative effects. Enmein specifically enhances VEGF expression and activates key signaling pathways such as phospho-Akt, p-GSK3β, and β-catenin, driving the proliferation of HFDPCs via the Akt/GSK3β/β-catenin transduction pathway. These properties underscore enmein's potential as a natural compound for treating AA [56].

#### 3.1.8 Loliolide

Loliolide is a metabolite derived from carotenoids, possessing characteristics of a potential endogenous inducer of herbivory resistance [74]. Biochemical tests revealed that low doses of loliolide boosted the viability and size of 3D DP spheroids in a dose-related way. This was linked to increased expression of hair growth-related factors like VEGF, IGF-1, and keratinocyte growth factor (KGF). Additional tests showed that loliolide induced Akt phosphorylation, stabilizing β-catenin crucial for hair-inductive properties in DPCs. It also enhanced the expression of DP signature genes and promoted keratinocyte proliferation and hair growth gene expression [57]. By promoting spheroid growth, upregulating hair growth factors, stabilizing β-catenin through Akt phosphorylation, and boosting DP signature gene expression, loliolide demonstrates potential as a therapeutic compound for treating AA.

#### 3.1.9 Lycopene

Lycopene, a dark red C_40_ terpenoid carotenoid found in tomatoes, watermelons, red carrots, and papayas, is a non-provitamin A compound recognized for its potent antioxidant properties and significant anticancer activity [75]. Lycopene offers various health benefits and has been extensively studied for its bioactive effects. A study investigating the hair-growth effects of *Lycopersicon esculentum* extracts in C57BL/6 mice found that isolated lycopene Tween-80 solution (LTS) and test hair tonic (THT) promoted hair growth comparable to 3% minoxidil. LTS increased VEGF, KGF, and IGF-1, while THT raised IGF-1 and reduced VEGF and TGF-β. Histology showed faster anagen induction with THT. Crucially, Draize tests confirmed non-irritant properties. Based on its ability to stimulate key hair growth factors, accelerate anagen induction, and exhibit a favorable safety profile, lycopene demonstrates significant potential as a natural therapeutic compound for AA [58].

#### 3.1.10 Cucurbitacins

Cucurbitacins, highly oxidized tetracyclic triterpenoids, are primarily derived from cucurbitaceae plants and classified into 12 types with over 200 derivatives. They have been reported to inhibit key signaling pathways, including JAK-STAT3 signaling, mammalian target of rapamycin (mTOR), vascular endothelial growth factor receptor (VEGFR), Wnt/β-catenin, and mitogen-activated protein kinase (MAPK) [59]. In experiment, cucurbitacin promotes hair regeneration by accelerating the transition of HFs from the telogen to anagen phase in C57BL/6J mice. Topical application of cucurbitacin cream induced earlier darkening and hair growth compared to the control. Histological analysis confirmed earlier HF cycle progression, and molecular studies showed significantly reduced *Fgf1*8 mRNA and FGF18 protein levels in cucurbitacin-treated skin [60]. These findings demonstrate cucurbitacin's potential as a therapeutic compound for AA, acting through the promotion of anagen entry and suppression of Fgf18 expression.

#### 3.1.11 Araliadiol in plants

Araliadiol in plants is a kind of pentacyclic triterpenoid compound. Ivy diol has a variety of potential biological activities, such as anti-inflammatory, antibacterial, and anti-tumor effects. A study used an *in vitro* model of human HF stem cells and hDPCs to evaluate the hair-growth-promoting effect of araliadiol in plants. Multiple experiments were conducted to detect its stimulation on cell proliferation and explore the mechanism. The results showed that araliadiol can stimulate hair growth by activating the p38 MAPK/peroxisome proliferator-activated receptor-γ (PPAR-γ) signaling pathway in human hair follicle stem cells and DPCs [61]. The efficacy of araliadiol is primarily attributed to this dual mechanism—stimulating proliferation in essential HF cell types and upregulating critical molecular regulators of the hair growth cycle. Therefore, these findings identify araliadiol as a promising therapeutic candidate for AA.

### 3.2 Flavonoids

Flavonoids, bioactive compounds abundant in foods such as tea, red wine, fruits, and vegetables, exhibit a wide array of physiological and pharmacological activities. Flavonoids are a major subclass of polyphenols. This review defines polyphenols as flavonoids and non-flavonoid phenolics, presenting the major flavonoid subclass in a dedicated section for clarity, with non-flavonoid phenolics discussed as a group. Renowned for their antioxidant properties and ability to chelate metal ions, flavonoids play significant roles in maintaining health and wellness [76,77]. Among the numerous approaches being investigated for AA treatment, flavonoid compounds have garnered increasing attention from the scientific community, owing to their promising biological activity and safety profile. These compounds, whether naturally occurring or synthetically derived, exhibit unique chemical structures and have demonstrated significant efficacy in modulating immune responses, enhancing hair growth, and inhibiting inflammation (Table 2) [[78], [79], [80], [81], [82], [83], [84], [85], [86], [87], [88]]. Flavonoids combat AA through several convergent mechanisms, and their shared actions include immunomodulation via inhibition of key inflammatory pathways like IFN-γ/JAK-STAT and CD8^+^ T-cell activity, coupled with the direct activation of crucial pro-growth signaling axes such as Wnt/β-catenin and Akt/ERK within DPCs. Furthermore, they universally promote HF cell proliferation and health by upregulating growth factors, enhancing cellular energy metabolism, and providing potent anti-apoptotic effects by modulating Bcl-2/Bcl-2-associated X protein (Bax) ratios and caspases. This multifaceted strategy effectively targets the core pathology of AA by rebalancing the immune response, restoring disrupted follicular signaling, and safeguarding HF cells from death, thereby collectively fostering an environment conducive to hair regeneration.

**Table 2:** Flavonoids with therapeutic potential for alopecia areata.

Compounds	Subclass	Sources	Mechanisms	Observed efficacy	Stage of evidence	Experimental models	Chemical construction	Refs.
Epigallocatechin gallate	Flavonoids	Green tea	• Inhibit STAT-1/JAK2/IRF-1 pathway • Increase the Bcl-2/Bax ratio, promoting DPC proliferation and anti-apoptosis • Upregulate phosphorylated Erk and Akt	Promote proliferation and survival of hDPCs and improve HF growth *in vitro*	Preclinical (*in vitro*)	hDPCs		[78,79]
			• Inhibit the expression of HLA-DR and HLA-B molecules via the IFN-γ pathway, suppress STAT-1/JAK2/IRF-1 pathway • Reduce CD8^+^NKG2D^+^ subset	Improve AA-related immune modulation in preclinical models	Preclinical (*ex vivo human evidence*, *in vitro*)	AA patient Peripheral Blood Mononuclear Cells (PBMCs), HaCaT cell		
Genistein	Isoflavone	Soybean	• Modulate estrogen-dependent mechanisms and/or inflammatory activity	Reduce AA incidence in C3H/HeJ mice	Preclinical (*in vivo*)	C3H/HeJ mice		[80]
Isoflavones	Isoflavone	Soybean	• Increase IGF-1 production in the HFDPCs in mice through increasing CGRP production in the sensory neurons	Enhance hair regeneration and pigmentation in mice	Preclinical (*in vivo*, *in vitro*)	WT C57BL/6 mice, CGRP-knockout (129/Sv-C57BL/6 hybrid) mice, primary DRG neurons (cultured)		[81,82]
Isoquercitrin	Flavonol glycoside	*Gossypium herbaceum L.*	• Induce autophagy via the AMPK/mTOR/ULK1 axis • Enhance angiogenesis through the IGF-1R/PI3K/Akt–VEGF pathway	Accelerate anagen entry and improve hair density and vascularization in rats	Preclinical (*in vivo*)	Sprague–Dawley rats		[83]
Silibinin	Flavonolignan	*Silybum marianum*	•Activate Akt and boost Wnt/β-catenin pathways	Increase 3D hDPC spheroid growth and viability and promote follicular induction	Preclinical (*in vitro*)	3D culture of DP cells		[84]
Sophoricoside	Isoflavone glycoside	*Sophora japonica* L.	•Through M4 mAChR to activate Wnt/β-catenin signaling	Promote *ex vivo* HF elongation and improve follicular cycling	Preclinical (*ex vivo*)	Cultured *ex vivo* mouse vibrissae HFs		[85]
Quercitrin	Flavonol glycoside	Vegetables and fruits	•Increase cell viability, energy metabolism, and growth factor expression (bFGF, KGF, VEGF) •Activate Akt, Erk, and CREB signaling	Increase hDPC viability and growth factor expression and improved hair shaft elongation	Preclinical (*in vitro*)	hDPCs		[86]
Formononetin	O-methylated isoflavone	Legumes and clovers	•Inhibit apoptosis by modulating caspase-8, procaspase-9, Bcl-2, Bax, and p53	Restore miniaturized follicles and inhibit apoptosis in C57BL/6 mice showing visible hair regrowth	Preclinical (*in vivo*)	C57BL/6 mice		[87]
Vitexin	Flavone glycoside	Vitex	•Augment the Wnt/β-catenin pathway	Stimulate hDPC proliferation and HF growth	Preclinical (*in vitro*)	hDPCs		[88]

STAT1: signal transducer and activator of transcription 1; JAK2: Janus kinase 2; IRF-1: interferon regulatory factor 1; Bcl-2: B-cell lymphoma 2; Bax: Bcl-2 associated X protein; DPC: dermal papilla cell; ERK: extracellular signal-regulated kinase; p-ERK: phosphorylated ERK; Akt: protein kinase B; hDPC: human dermal papilla cell; hDPCs: human dermal papilla cells; HF: hair follicle; HFs: hair follicles; HLA-DR: human leukocyte antigen-DR; HLA-B: human leukocyte antigen-B; IFN-γ: interferon-gamma; CD8^+^: cluster of differentiation 8 positive; NKG2D: natural killer group 2, member D; HaCaT: human adult low calcium high temperature keratinocytes; PBMCs: peripheral blood mononuclear cells; AA: alopecia areata; HFDPCs: hair follicle dermal papilla cells; IGF-1: insulin-like growth factor 1; CGRP: calcitonin gene-related peptide; AMPK: AMP-activated protein kinase; mTOR: mammalian target of rapamycin; ULK1: Unc-51 like autophagy activating kinase 1; IGF-1R: insulin-like growth factor 1 receptor; PI3K: phosphoinositide 3-kinase; VEGF: vascular endothelial growth factor; Wnt: wingless-related integration site; β-catenin: beta-catenin; M4 mAChR: muscarinic acetylcholine receptor M4; bFGF: basic fibroblast growth factor; KGF: keratinocyte growth factor; CREB: cAMP response element-binding protein; p53: tumor protein p53.

#### 3.2.1 Epigallocatechin gallate (EGCG)

The compound EGCG, classified as a flavonoid, is a polyphenolic catechin that constitutes approximately 59% of the overall catechins present in green tea leaves [89]. A study evaluated EGCG as a safer, topically active inhibitor of IFN-γ signaling in alopecia areata using HaCaT and Jurkat cells and peripheral blood mononuclear cells (PBMCs) from 30 patients and 30 healthy controls. EGCG abolished STAT1 phosphorylation after 48 h, likely via selective JAK2 downregulation, which decreased HLA-DR and HLA-B expression. Although AA PBMCs showed elevated T helper 1 (Th1), Th17, and CD8 responses with higher IFN-γ and IL-17, EGCG did not change total CD8 levels but significantly reduced the pathogenic CD8^+^ NKG2D^+^ subset [78].

In parallel, studies on hDPCs show that EGCG activates proliferative signaling through ERK and Akt phosphorylation and exerts anti-apoptotic effects by increasing the Bcl-2/Bax ratio, collectively stimulating human hair growth [79]. Collectively, *in vitro* and *ex vivo* findings support EGCG as a promising preclinical candidate with translational potential for AA therapy.

#### 3.2.2 Genistein

Genistein, a major active component of soybean isoflavones, exerts estrogen-like functions and has demonstrated various biological effects in preclinical studies. These include antioxidant, anti-inflammatory, antibacterial, and antiviral activities, as well as effects on angiogenesis, estrogen modulation, diabetes, and lipid metabolism [90].

Experimental evidence indicates that genistein may protect against AA development via two primary mechanisms: modulation of estrogen pathways and regulation of inflammatory responses. In C3H/HeJ mouse models, dietary intervention demonstrated a clear dose dependent preventive effect. Supplementation with soy oil at concentrations of 1%, 5%, and 20% led to AA incidence rates of 86%, 39%, and 18%, respectively. More notably, the combination of subcutaneous genistein administration and a 1% soy oil diet reduced AA occurrence to 40%, compared to 90% in the control group [80].

Based on its direct effects observed in AA model mice, genistein shows potential to prevent hair loss through coordinated regulation of the endocrine and immune systems. This compound's multimodal activity combines effects similar to estrogen with anti-inflammatory properties, suggesting it can intervene in AA progression at multiple pathological levels.

#### 3.2.3 Isoflavones

The main dietary sources of isoflavones for humans are legumes from the family Fabaceae [91]. In a study of the hypothesis using wild-type (WT) and CGRP-knockout mice, isoflavone significantly increased the *Cgrp* mRNA levels in dorsal root ganglion (DRG) neurons isolated from WT mice. After 3 weeks, isoflavone also increased dermal CGRP and IGF-1 levels and elevated *Igf1* mRNA in WT mice, whereas these effects were not observed in CGRP-knockout mice. Consistently, isoflavone administration increased immunohistochemical IGF-1 expression in WT mice. Significant enhancements of HF morphogenesis, hair regrowth, and hair pigmentation were also observed in WT mice administered isoflavone. However, none of these effects in HFDPCs in WT mice were observed in CGRP-knockout mice. This demonstrates that isoflavone promotes hair growth and associated melanogenesis in mice, potentially by increasing CGRP production in sensory neurons to boost IGF-1 in HFDPCs, thereby establishing its therapeutic potential for AA through the reactivation of follicular regeneration and pigmentation via this signaling pathway [81,82].

#### 3.2.4 Isoquercitrin

Isoquercitrin, a flavonol glycoside, is known for anti-aging properties. Recent studies have demonstrated its efficacy in promoting hair growth *in vivo* and have shown that it acts through regenerative pathways relevant to AA. In middle-aged female Sprague-Dawley (SD) rats, topical isoquercitrin accelerated anagen entry and regrowth, increasing hair density, length, growth rate, and weight, while hematoxylin and eosin (H&E) histology showed more and longer anagen follicles with greater skin thickness and visibly denser dermal vasculature.

Mechanistically, target-engagement assays such as cellular thermal shift assay (CETSA), drug-affinity responsive target stability (DARTS) and surface plasmon resonance (SPR) show direct binding to AMP-activated protein kinase (AMPK) and insulin-like growth factor-1 receptor (IGF-1R) and suggest that this engagement activates two coordinated pathways. One pathway involves AMPK activation together with mTOR inhibition and unc-51-like autophagy-activating kinase 1 (ULK1) phosphorylation, which enhances autophagic flux as reflected by increased microtubule-associated protein 1 light chain 3-II (LC3-II) and decreased sequestosome-1 (p62). The other pathway involves IGF-1R phosphorylation that activates downstream PI3K-Akt signaling, upregulates VEGF and angiotensin (ANG), and increases phosphorylation of VEGFR1 and VEGFR2, thereby promoting angiogenesis and improving tissue perfusion [83].

#### 3.2.5 Silibinin

Silibinin is a natural polyphenolic flavonoid that is present in seed extracts of milk thistle (*Silybum marianum* (L.) Gaertn.). It exhibits a broad range of beneficial effects, including antioxidant, antineoplastic, hepatoprotective, neuroprotective, anti-inflammatory, antimicrobial, and antidiabetic properties [92]. A study found silibinin significantly enhances 3D human DP spheroid viability and growth via Akt activation, which promotes proliferation and hair induction through the Akt and Wnt/β-catenin signaling pathways in 3D DP spheroids [84]. These findings establish silibinin as a compelling therapeutic candidate for AA by targeting the reactivation of the dysregulated Akt and Wnt/β-catenin signaling pathways, a core molecular axis that governs HF cycling, DP cell functionality, and follicular regeneration.

#### 3.2.6 Sophoricoside (SOP)

SOP is an isoflavone glycoside isolated from the seed of the medicinal herb *Sophora japonica* L. A study aimed to determine the ability of Fructus Sophorae extract and SOP to promote hair growth and its signaling mechanism through molecular docking, cell line studies, and *ex vivo* HF cultures. Results indicated that SOP promoted hair growth in cultured *ex vivo* mouse vibrissa HF by acting through M4 muscarinic acetylcholine receptor (M4 mAChR) to activate Wnt/β-catenin signaling [85]. These findings establish SOP as a novel therapeutic candidate for AA, primarily through its targeted activation of the M4 mAChR to Wnt/β-catenin signaling axis—a key mechanism for restoring dysregulated HF cycling in autoimmune hair loss.

#### 3.2.7 Quercitrin (Qc)

Qc is a naturally occurring flavonoid commonly found in dietary supplements, known for its broad spectrum of bioactivities. It exhibits antioxidative, anti-inflammatory, antimicrobial, immunomodulatory, analgesic, wound-healing, and vasodilatory effects, and has been shown to exert significant anti-inflammatory effects in various diseases. A study explored Qc's hair growth effects in hDPCs. It increased cell viability, energy metabolism, and the expression of growth factors such as basic fibroblast growth factor (bFGF), KGF, and VEGF. This was accompanied by the activation of key signaling molecules, namely Akt, ERK, and cAMP response element-binding protein (CREB). It also promoted hair shaft growth. These findings establish Qc as a promising therapeutic candidate for AA, primarily through its unique capacity to simultaneously counteract follicular inflammation, restore DP cell bioenergetics, and reactivate the MAPK-CREB signaling pathway [86].

#### 3.2.8 Formononetin (FORM)

FORM is an O-methylated isoflavone and a member of the phytoestrogen group, commonly found in legumes and various types of clovers, such as *Trifolium pratense* L., *Astragalus membranaceus*, and *Sophora tomentosa*. It exhibits broad pharmacological activities including anticancer, anti-apoptotic, anti-inflammatory, and antioxidative effects mediated through estrogen-dependent/independent pathways [93]. In depilated C57BL/6 mice, topical FORM application for 14 days significantly promoted hair regrowth, restored miniaturized HFs, and inhibited apoptosis by modulating caspase-8, pro-caspase-9, Bcl-2, Bax, and p53. Terminal deoxynucleotidyl transferase dUTP nick end labeling (TUNEL) staining confirmed reduced apoptosis. Overall, FORM's hair regrowth effect is linked to caspase regulation and apoptosis inhibition [87]. These findings establish FORM as a potent therapeutic candidate for AA, primarily through its targeted suppression of apoptotic signaling cascades—a core pathological driver of autoimmune-mediated HF destruction.

#### 3.2.9 Vitexin

Vitexin, a flavone glycoside derived from apigenin, naturally occurs in both food and medicinal plants. It exhibits a diverse range of pharmacological properties, encompassing antioxidant, anti-inflammatory, anticancer, analgesic, and neuroprotective activities [94]. To evaluate its pro-regenerative potential, vitexin compound 1 (VB-1) was applied to hDPCs, and cell proliferation, hair-growth-related gene and protein expression, and HF growth were assessed. VB-1 increased hDPC proliferation and promoted HF growth. Mechanistically, VB-1 facilitated Wnt/β-catenin signaling, evidenced by Dickkopf-related protein 1 (DKK1) downregulation, β-catenin accumulation, and decreased axis inhibition protein 2 (AXIN2). Taken together, this pro-regenerative activity and clear pathway engagement support VB-1 as a promising therapeutic candidate for AA [88].

### 3.3 Non-flavonoid polyphenols

Polyphenols are secondary plant metabolites with antioxidant, anti-inflammatory, and anti-microbial activity. They are ubiquitously distributed in the plant kingdom, and contain high amounts, such as green tea and grape seeds [95]. Polyphenols, particularly the non-flavonoid subclass are attractive ingredients for pharmacy due to their beneficial biological properties, including hair growth (Table 3) [[96], [97], [98], [99]]. The therapeutic efficacy of non-flavonoid polyphenols like curcumin, resveratrol, and salvianolic acid B in AA stems from their shared ability. Their similarities lie in their ability to modulate the HF cycle by promoting the active growth phase and delaying the regression phase, protect hDPCs from oxidative stress-induced damage, and suppress the autoimmune inflammation that drives follicular attack, with curcumin specifically blocking the key p21-activated kinase 1 (PAK1) signaling pathway. Furthermore, they promote the proliferation and viability of essential follicular cells. This combination of regulating follicular cycling, providing antioxidant protection, and exerting anti-inflammatory actions establishes a common rationale for their use in treating AA.

**Table 3:** Non-flavonoid polyphenols with therapeutic potential for alopecia areata.

Compounds	Subclass	Sources	Mechanisms	Observed efficacy	Stage of evidence	Experimental models	Chemical construction	Refs.
Curcumin	Polyphenols	Turmeric	• Inhibit PAK1	Promote HF regeneration and show positive effect in AA when combined with piperine and capsaicin	Preclinical (with preliminary human evidence)	Human patients		[96,97]
Resveratrol	Polyphenols	Plants, including grapes, peanuts, and berries	• Enhance the proliferation of hDPCs	Induce anagen entry and delay catagen transition in C57BL/6 mice and improve hDPC proliferation	Preclinical (*in vivo*, *in vitro*)	C57BL/6 mice, hDPCs		[98]
Salvianolic Acid B	Phenolic acid	*Salvia miltiorrhiza*	• Protect against H_2_O_2_-induced oxidative damage • Reduce ERK and p38 phosphorylation	Promote angiogenesis and prolonged anagen phase in mouse and human HF models	Preclinical (*in vivo*, *ex vivo*, *in vitro*)	C57BL/6 mice, human HFs, hDPCs		[99]

PAK1: p21-activated kinase 1; HF: hair follicle; AA: alopecia areata; hDPCs: human dermal papilla cells; ERK: extracellular signal-regulated kinase; p38: p38 mitogen-activated protein kinase.

#### 3.3.1 Curcumin

Curcumin, a lipophilic polyphenol and bioactive compound in turmeric, has gained attention for its anticancer, anti-inflammatory, and anti-aging properties. Recent studies highlight its potential in treating skin conditions, including inflammatory and neoplastic diseases. Despite bioavailability challenges, curcumin shows promise as a therapeutic agent for skin health [100,101]. Herbal PAK1-blockers curcumin from *Curcuma longa* L. have been shown to be anti-oncogenic and anti-melanogenic as well as anti-alopecia [96]. Clinically, formulations combining curcumin with piperine and capsaicin show efficacy in treating AA [97]. These collective properties establish curcumin as a promising therapeutic candidate for AA primarily through its blockade of PAK1 signaling, a key driver of autoimmune-mediated follicular inflammation and miniaturization in AA pathogenesis.

#### 3.3.2 Resveratrol (RE)

RE, a polyphenolic compound belonging to the stilbene class, is found in a variety of plants, including grapes, peanuts, and berries. Known for its wide array of health benefits, RE has gained attention for its potential in combating obesity, protecting the heart and brain, preventing cancer, managing diabetes, acting as an antioxidant, slowing aging, and regulating glucose metabolism [102].

The topical application of RE notably accelerated hair growth and triggered the shift from the telogen phase to the anagen phase in shaved C57BL/6 mice. *Ex vivo* results revealed that RE promoted hair shaft elongation in HFs and postponed the transition into the catagen phase. *In vitro* studies also demonstrated that RE enhanced the proliferation of hDPCs and protected them from oxidative stress induced by hydrogen peroxide (H_2_O_2_). RE exhibits the ability to stimulate hair growth and holds promise as a potential therapeutic option for addressing hair loss [98]. The combination of anagen phase activation, hair cycle modulation, and enhanced oxidative stress resistance in hDPCs establishes RE as a compelling therapeutic candidate for AA by directly counteracting both dysregulated follicular cycling and cellular damage in autoimmune mediated hair loss.

#### 3.3.3 Salvianolic acid B (SalB)

SalB is a monomeric component of *Salvia miltiorrhiza*, which has antioxidant and anti-inflammatory influences that are linked to hair loss. Experiments using mice revealed that SalB treatment stimulated new hair growth and improved blood vessel formation around HFs within 21 days. When tested on human hair samples, SalB not only increased hair length by 37% but also prolonged the active growth phase while delaying natural hair regression.

Cell-based studies demonstrated that SalB enhanced the proliferation of key HF cells by over 40%, protected them from H_2_O_2_-induced oxidative damage, and reduced activation of stress-related proteins, particularly ERK and p38 MAPK [99]. Collectively, the combined enhancement of angiogenesis, regulation of HF cycling, and protection against oxidative stress and ERK/p38-mediated stress responses positions SalB as a promising therapeutic candidate for AA.

### 3.4 Other compounds

Several other compounds, though less frequently discussed, have demonstrated promising potential for treating AA. These therapeutic agents, including azelaic acid, amygdalin, and cepharanthine, offer unique mechanisms of action and potential benefits in managing AA. Due to the limited number of studies on each of these compounds, they are grouped together for a more comprehensive discussion (Table 4) [[103], [104], [105], [106], [107], [108], [109], [110]].

**Table 4:** Other compounds with therapeutic potential for alopecia areata.

Compounds	Subclass	Sources	Mechanisms	Observed efficacy	Stage of evidence	Experimental models	Chemical construction	Refs.
Azelaic acid	Dicarboxylic acid	Grain	• Increase catalase activity • Upregulate Gli1/Gli2	Promote hair regrowth comparable to dithranol and show synergy with minoxidil	Clinical	Clinical patients		[103]
Amygdalin	Cyanogenic glycoside	Bitter almonds	• Inhibit the JAK2/STAT3 signaling pathway	Improve hair regrowth and reduce inflammation in AA mice	Preclinical (*in vivo*)	C3H/HeJ mice		[104,105]
*Ginkgo biloba* leaves polysaccharides	Polysaccharides	*Ginkgo biloba*	• Downregulate the expression of p-p65, p-IκBα, TNF-α, and IL-1β proteins in the inflammation signaling pathway	Promote hair regrowth and reduce perifollicular inflammation in AA mouse models	Preclinical ( *in vivo*, *in vitro*)	C57BL/6 mice, HUVECs	N/A	[[106], [107], [108]]
Deoxyshikonin	Naphthoquinone	Arnebiae Radix	• Activate Wnt/β-catenin signaling pathway • Upregulate β-catenin and p-GSK3β, increasing the expression of VEGF and IGF-1	Enhance HF regeneration in AA mice	Preclinical (*in vivo*, *in vitro*)	C57BL/6 mice, hDPCs		[109]
Cepharanthine	Alkaloid	Stephania cepharantha Hayata	• Induce the expression of VEGF and modulate associated signaling pathways	Promote the proliferation of hDPCs and hORSCs and increase VEGF expression	Preclinical (*in vitro*)	hDPCs, hORSCs		[110]

Gli1: glioma-associated oncogene homolog 1; Gli2: glioma-associated oncogene homolog 2; JAK2: Janus kinase 2; STAT3: signal transducer and activator of transcription 3; AA: alopecia areata; p-p65: phosphorylated p65; p-IκBα: phosphorylated inhibitor of kappa B alpha; TNF-α: tumor necrosis factor-alpha; IL-1β: interleukin-1 beta; HUVECs: human umbilical vein endothelial cells; N/A: not applicable; Wnt: wingless-related integration site; β-catenin: beta-catenin; p-GSK3β: phosphorylated glycogen synthase kinase 3 beta; VEGF: vascular endothelial growth factor; IGF-1: insulin-like growth factor 1; hDPCs: human dermal papilla cells; hORSCs: human outer root sheath cells.

#### 3.4.1 Azelaic acid

Azelaic acid, a naturally occurring and non-toxic C_9_ dicarboxylic acid, possesses significant biological properties and potential as a therapeutic agent [111].

Clinical investigations have demonstrated its potential in AA management. In a comparative 12-week trial involving 31 patients with patchy AA, topical 20% azelaic acid exhibited comparable efficacy to 0.5% dithranol in promoting hair regrowth, with both treatments maintaining favorable safety profiles [103]. While these preliminary results are encouraging, further large-scale clinical validation is warranted.

Beyond its clinical performance, mechanistic studies reveal azelaic acid's unique capacity to act synergistically with minoxidil. While minoxidil primarily upregulates Gli1 and Axin2, azelaic acid demonstrates distinct activity by enhancing catalase activity and modulating Gli1/Gli2 pathways. Their combination produces a synergistic effect through significant upregulation of sonic hedgehog (Shh) protein expression [112]. Crucially, its direct action in AA-related models, through this dual mechanism that promotes HF regeneration and provides photoprotection against ultraviolet B (UVB)-induced damage, underscores its considerable and comprehensive therapeutic potential for AA.

#### 3.4.2 Amygdalin

Amygdalin is a cyanogenic glycoside found in the seeds of plants like bitter almonds and peaches. It has shown pharmacological activities such as anti-tumor, anti-inflammatory, analgesic, and immunomodulatory effects, and benefits for cardiovascular, digestive, reproductive, and neurological health [113].

Xuefu Zhuyu Decoction (XZD) has been shown to significantly alleviate AA-related skin damage induced by chronic unpredictable mild stress (CUMS) in C3H/HeJ mice. This protective effect is potentially achieved through the suppression of IL-6, IL-1β, TNF-α, and osteopontin levels. XZD could visibly promote hair regeneration in AA patients. The potential active ingredients in the XZD prescription included amygdalin [104]. Directly investigating amygdalin for AA treatment in mice, administration improved skin pathology and hair growth. It significantly reduced pro-inflammatory cytokines in serum and skin, while rebalancing immune cells. Crucially, amygdalin exerted its effects by inhibiting the JAK2-STAT3 pathway activation and elevating suppressor of cytokine signaling 3 (SOCS3) [105].

Thus, amygdalin alleviates AA by inhibiting inflammation, restoring immune balance, and blocking the JAK2-STAT3 pathway, supporting its role in XZD's therapeutic effects. The demonstrated efficacy of amygdalin in AA models through its multi-faceted action targeting inflammation and a key signaling pathway confirms its strong standalone therapeutic potential.

#### 3.4.3 *Ginkgo biloba* leaves polysaccharides

For thousands of years, *Ginkgo biloba* L. has been recognized as a highly promising source of both food and traditional herbal remedies. As far back as the previous century, Japanese researchers discovered that *Ginkgo biloba* could stimulate hair regrowth in mice after shaving the back of their fur. An investigation was conducted to assess the impact of a 70% ethanolic extract derived from *Ginkgo biloba* leaves, referred to as Ginkgo biloba extract (GBE), on hair regrowth in C3H strain mice whose posterior hair had been shaved. The findings revealed that GBE exhibited a stimulatory effect on hair regrowth. Additionally, GBE demonstrated inhibitory effects on blood platelet aggregation, thrombin activity, and fibrinolysis. These results show GBE's potential to promote hair regrowth, suggesting its viability as a hair tonic [106].

Recent research has further elucidated the therapeutic potential of *Ginkgo biloba* polysaccharides, particularly their water-soluble fractions. As primary bioactive constituents, these polysaccharides demonstrate multifaceted biological activities including antioxidant, immunomodulatory, and anti-inflammatory effects, with emerging evidence supporting their role in HF stimulation [107]. A study about water-soluble *Ginkgo biloba* leaves polysaccharides (WGBP) was conducted to observe the hair-growth enhancing effects in mice with AA, to investigate its anti-inflammatory mechanism. This study demonstrates that *Ginkgo biloba* acidic polysaccharides, particularly the purified fraction WGBP-A2b, alleviate perifollicular inflammation and promote hair regrowth.

Mechanistically, WGBP-A2b modulated inflammatory responses, upregulated VEGF and hepatocyte growth factor (HGF), and inhibited NF-κB signaling. At the cellular level, WGBP-A2b treatment significantly downregulated the expression of phosphorylated-p65 (p-p65), phosphorylated-IκBα (p-IκBα), TNF-α, and IL-1β in HUVECs, suggesting its anti-inflammatory effects are mediated through modulation of these key proteins in the NF-κB signaling pathway [108].

Building upon centuries of traditional use and modern scientific validation, WGBP emerges as a highly promising natural candidate for AA therapy. Critically, its direct and mechanistic efficacy in established AA models, mediated through NF-κB pathway suppression and the promotion of key growth factors, solidifies its considerable potential for development into a novel therapeutic agent.

#### 3.4.4 Deoxyshikonin (Ds)

Ds is a naphthoquinone derivative isolated from Arnebiae Radix (AR), a traditional Asian medicinal herb. Recent investigations identified Ds as the primary bioactive component of AR responsible promoting hair growth. Preclinical studies employing C57BL/6 mouse with AA demonstrated that Ds significantly enhanced hair regeneration and improved DPC proliferation. Mechanistic investigations revealed that Ds directly targets GSK3β, leading to the activation of the Wnt/β-catenin signaling pathway—a key regulator of HF cycling and regeneration [109]. Functionally, Ds upregulated β-catenin and p-GSK3β, and increased the expression of VEGF and IGF-1, while downregulating TGF-β1, thereby creating a favorable microenvironment for HF growth.

Collectively, these findings highlight Ds as a promising natural therapeutic candidate for AA, exerting its effects predominantly through modulation of the Wnt/β-catenin pathway. Its direct efficacy in an AA model, achieved by precisely targeting the GSK3β-mediated signaling axis, strongly underscores its translational potential as a targeted treatment for the condition.

#### 3.4.5 Cepharanthine (CEP)

CEP, a bisbenzylisoquinoline alkaloid, is isolated and extracted from the Menispermaceae plant, *Stephania cepharantha* Hayata. It boasts various pharmacological activities, encompassing antioxidant, anti-inflammatory, immunomodulatory, antitumor, and antiviral benefits [114]. This research investigated the impact of CEP and its derivatives on hDPCs and human outer root sheath cells (hORSCs). The findings revealed that CEP and certain analogues were capable of stimulating the growth of hDPCs, inducing the expression of VEGF, and modulating associated signaling pathways. These results suggest that CEP and its analogues possess potential hair growth-promoting properties, offering novel insights into hair regeneration research [110]. These findings position CEP as a promising therapeutic candidate for AA, primarily through its dual capacity to stimulate follicular cell growth via VEGF induction and regulation of immune responses.

## 4 Natural formulations with therapeutic potential for AA

Beyond single-compound agents, complex natural formulations provide multi-component synergy that offers therapeutic advantages against the multifactorial pathogenesis of AA and are discussed in this review as promising therapeutic supplements. The multifaceted mechanisms of action, coupled with their favorable safety profiles, have positioned these natural products as valuable components in integrative treatment approaches for AA and other hair loss disorders, attracting increasing attention in both clinical research and therapeutic applications.

### 4.1 *Centipeda minima* extract (CMX)

*Centipeda minima*, a plant from the Asteraceae family native to eastern tropical Asia, has medicinal properties traditionally used to treat conditions like whooping cough, asthma, and malaria. Recent research has shown that CMX can inhibit inflammation and cell proliferation by regulating the JAK-STAT signaling pathway in macrophages and keratinocytes. This highlights its potential for treating inflammatory and autoimmune diseases [115].

CMX, including the CMX-derived compound DN106212, promotes hair growth through multiple mechanisms. In human HFDPCs, CMX upregulates the Wnt/β-catenin, ERK, and c-Jun N-terminal kinase (JNK) signaling pathways, increasing the expression of growth factors like VEGF and IGF-1, which are linked to hair regeneration [116]. Similarly, in C57BL/6 mice, DN106212 outperformed dimethyl sulfoxide and tofacitinib in promoting hair growth over 16 days, as shown by quantitative polymerase chain reaction (qPCR), which revealed increased *Vegfa* and *Igf1* expression. Inhibition of TGF-β1 had effects similar to tofacitinib treatment, suggesting that DN106212 may enhance hair growth by modulating growth factors. Computational analysis indicates CMX compounds have good bioavailability, supporting their potential as a natural treatment for hair loss. Further research is needed to fully explore their therapeutic benefits [117]. CMX and DN106212 emerge as a promising AA treatment by dual activation of Wnt/β-catenin mediated hair regeneration and suppression of JAK-STAT driven inflammation and TGF-β1 related fibrosis, thereby comprehensively addressing the disease pathology.

### 4.2 Black tea extract

Black tea constitutes 70%–80% of global tea production, with its polyphenols resulting from the enzymatic oxidation of four tea catechins during fermentation. Chinese black tea extract (CBTE), fermented with Aspergillus species, significantly promoted hair growth in 6-week-old male C3H/HeJ mice after 2 weeks of topical application [118]. Mechanistic investigations identified CBTE's selective binding affinity for estrogen receptor α (ERα), with an half-maximal inhibitory concentration (IC_50_) value of 74.8 μg/mL, suggesting ER-mediated pathways analogous to soy isoflavone—a known phytoestrogen. Notably, this activity appears potentiated by capsaicin, indicating potential synergistic effects in combinatorial formulations [81]. CBTE exerts its therapeutic effects for AA through ERα-mediated follicular cycle regulation and enhanced vascularization via estrogenic pathways, with capsaicin potentially amplifying efficacy through transient receptor potential vanilloid 1 (TRPV1) activation, establishing it as a promising treatment candidate.

### 4.3 *Geranium sibiricum* L. extract (GSE)

*Geranium sibiricum* L. is a plant of the Geraniol seedling family and Geranium genus. GSE promotes hair growth both *in vitro* and *in vivo*. *In vitro* studies showed that GSE stimulates the proliferation and migration of hDPCs, and increases the expression of growth factors such as HGF and VEGF. *In vivo,* studies in shaved C57BL/6 mice demonstrated that topical application of GSE promoted significant hair growth compared with minoxidil. Additionally, GSE reduced inflammation by decreasing the number of mast cells and the expression of TGF-β1 in mouse skin tissues. Overall, these results suggest that GSE promotes hair growth by regulating growth factors and cellular responses [119]. These findings demonstrate GSE's potential for AA treatment by simultaneously promoting follicular regeneration via HGF/VEGF upregulation and suppressing inflammation through mast cell/TGF-β1 modulation.

### 4.4 *Betula platyphylla* extracts (BPE)

BPE demonstrates promising hair growth promotion through dual mechanisms, as revealed by recent research. The extract contains antioxidant phenolic compounds that enhance hair-growth-related gene expression and stimulate HF formation. Concurrently, BPE reduces inflammatory factor production while activating DPCs. These bioactive small molecules effectively regulate HF cycling through coordinated molecular pathways [120]. By coordinately regulating hair cycle progression through molecular pathway modulation, BPE demonstrates compelling potential as a therapeutic candidate for AA by addressing the proliferative impairment as well as the inflammatory pathogenesis central to autoimmune-mediated HF dystrophy.

### 4.5 Rosehip seed oil (RHSO)

RHSO, rich in bioactive compounds, demonstrates potential hair-growth-promoting effects in murine models. A 21-day topical application of RHSO on telogen-phase mice induced premature transition of HFs into the growth phase, enhanced follicular density, and upregulated hair-growth-associated gene expression [121]. These effects demonstrate RHSO's potential as a therapeutic candidate for AA through targeted modulation of hair cycle progression pathways to counteract autoimmune-related hair loss.

### 4.6 *Lagerstroemia indica* extract (LIE)

*Lagerstroemia indica*, belonging to the Lythraceae family, is a widely cultivated ornamental woody shrub or tree that holds significant importance as a traditional medicinal plant in both East Asia and Egypt [122]. *In vitro* and clinical studies found that LIE significantly stimulates the proliferative activity of HF cells, effectively halts the process of follicular degeneration, and demonstrates remarkable hair growth-promoting effects by finely regulating key signaling pathways such as Wnt-β-catenin, JAK3-STAT6, and TGF-β1-Smad. Clinical validation further supports its efficacy in halting hair loss [123]. These properties establish LIE as a promising therapeutic candidate for AA by simultaneously activating follicular regeneration pathways while suppressing inflammatory and fibrotic signaling implicated in autoimmune-mediated HF destruction.

### 4.7 *Miscanthus sinensis* var. *purpurascens* (MSP) flower extract

MSP, commonly known as flame grass, is native to Korea, Japan, and China, and possesses a range of biological activities such as anti-cancer properties, detoxification capabilities, vasodilatory effects, antipyretic actions, and diuretic functions [124]. Experimental investigation revealed that MSP flower extract stimulates HF activation through coordinated molecular regulation. Topical application significantly accelerated HF cycling by downregulating TGF-β1 while simultaneously upregulating HGF and β-catenin expression in murine models. This treatment induces premature telogen-to-anagen transition, enhances follicular stem cell factor production, and promotes morphological development of HFs, establishing MSP as a promising therapeutic candidate for AA [125].

### 4.8 Nelumbinis Semen extract

Nelumbinis Semen, a time-honored functional food and medicine, is rich in nutrients and bioactive compounds crucial for physiological functions, and it is renowned for its natural therapeutic properties and health benefits, such as antioxidant, anti-inflammatory, and cardiovascular protective effects [126]. Research has shown that Nelumbinis Semen extract possesses significant hair growth-promoting and hair loss-preventing effects. *In vitro*, the extract enhances cell proliferation and migration in a concentration-dependent manner, while exhibiting potent antioxidant activity. *In vivo*, it improves hair growth patterns in mice and upregulates the expression of growth factors related to hair growth. Therefore, Nelumbinis Semen extract, as a novel natural substance, holds great promise for promoting hair growth in AA [127].

### 4.9 Cacumen Platycladi (CP) water extract

CP consists of the dried needles of *Platycladus orientalis* (L.) Franco. A study investigated the hair growth-promoting mechanism of Cacumen Platycladi water extract (WECP) from *Platycladus orientalis*. Experiments revealed that WECP enhanced hair regeneration by stimulating DPC proliferation and migration while regulating cell cycle proteins. Mechanistic studies identified activation of the Akt/GSK3β/β-catenin pathway as critical, with WECP increasing β-catenin activity and promoting follicle development. The effects were reversed by Akt inhibitors, confirming pathway specificity [128]. These findings establish WECP as a promising therapeutic candidate for AA through targeted activation of the Akt/β-catenin axis, directly counteracting the suppressed Wnt signaling and impaired follicle regeneration central to autoimmune-mediated hair loss pathology.

### 4.10 Propolis

Propolis, a bioactive bee-collected resin with demonstrated antibacterial, antifungal, antiviral, and immunomodulatory properties [129], promotes hair growth through Wnt/β-catenin pathway activation. In C57BL/6N mice, Philippine stingless bee propolis promoted hair follicle development and dorsal-skin melanization, increasing *Wnt3a*, *Ctnnb1* (β-catenin), *Lef1*, and bone morphogenetic protein 2 (*Bmp2*) mRNA and elevating Wnt3a and β-catenin protein levels [130]. Ethanol-extracted propolis enriched with caffeic acid and kaempferol induces anagen phase transition, accelerates hair matrix keratinocyte proliferation and migration, and enhances epidermal keratinocyte growth without altering follicular architecture [131]. These mechanisms establish propolis as a promising therapeutic candidate for AA by reactivating Wnt-mediated follicular regeneration while leveraging its inherent immunomodulatory activity to counteract autoimmune-mediated HF destruction.

## 5 Discussion

**Fig. 3:**
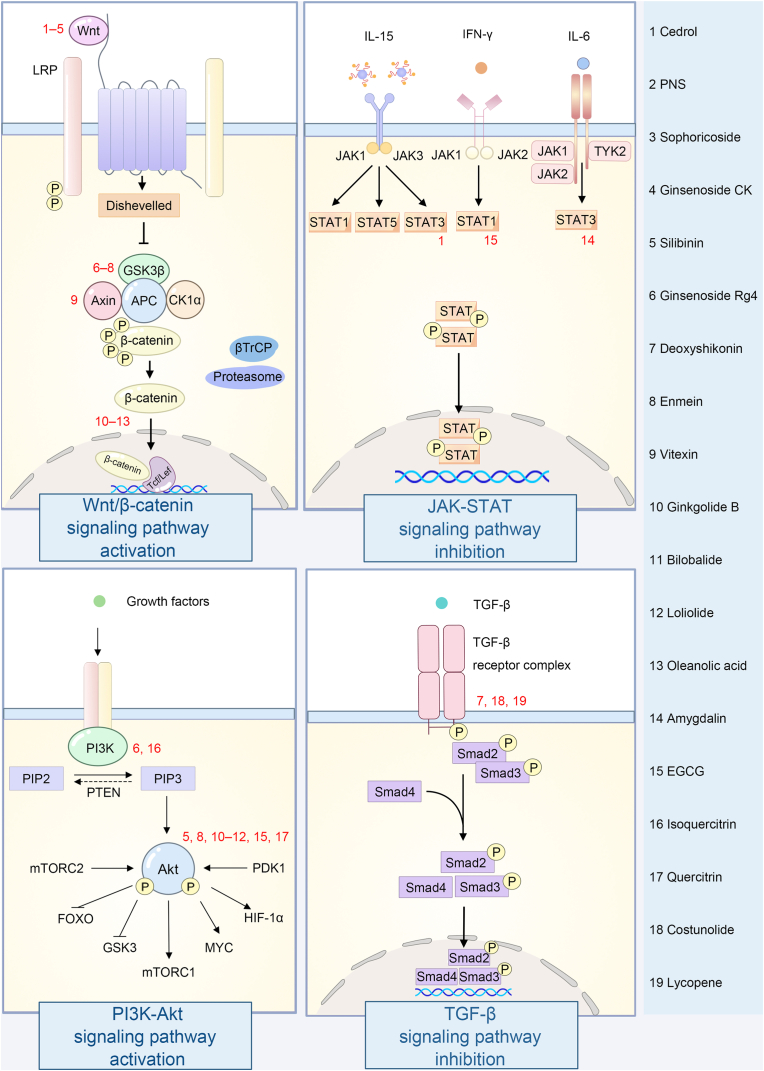
Mechanistic landscape of natural products in alopecia areata. Natural products act through complementary nodes that rebalance regeneration and immunity. They activate wingless/int-1 (Wnt)/β-catenin and phosphoinositide 3-kinase-protein kinase B (PI3K-Akt) programs to drive anagen entry, follicular proliferation, and microvascular support, while inhibiting Janus kinase-signal transducer and activator of transcription (JAK-STAT) signaling downstream of interferon-γ (IFN-γ) and interleukin-15 (IL-15) and tempering transforming growth factor-β (TGF-β)-mediated inflammatory cues to help restore hair follicle immune privilege. Because several compounds engage multiple nodes simultaneously, multi-target synergy is common, yielding coordinated effects that attenuate perifollicular autoimmunity and improve hair-cycle dynamics. APC: adenomatous polyposis coli; Axin: axis inhibition protein; β-TrCP: β-transducin repeat-containing protein; CK1α: casein kinase 1 α; FOXO: forkhead box O; GSK3β: glycogen synthase kinase 3β; HIF-1α: hypoxia-inducible factor 1-α; IL-6: interleukin-6; JAK1: Janus kinase 1; JAK2: Janus kinase 2; JAK3: Janus kinase 3; MYC: myelocytomatosis oncogene; PDK1: 3-phosphoinositide-dependent protein kinase 1; PIP2: phosphatidylinositol 4,5-bisphosphate; PIP3: phosphatidylinositol 3,4,5-trisphosphate; STAT1: signal transducer and activator of transcription 1; STAT3: signal transducer and activator of transcription 3; STAT5: signal transducer and activator of transcription 5; Tcf/Lef: T-cell factor/lymphoid enhancer factor; mTORC1: mechanistic target of rapamycin complex 1; mTORC2: mechanistic target of rapamycin complex 2; Smad2: mothers against decapentaplegic homolog 2; Smad3: mothers against decapentaplegic homolog 3; Smad4: mothers against decapentaplegic homolog 4.

In recent years, natural products have shown therapeutic promise for AA by modulating an extensive signaling network that encompasses the Wnt/β-catenin, JAK-STAT, PI3K-Akt, TGF-β, ERK, p38 MAPK, NF-κB, NOD-like receptor family pyrin domain-containing 3 (NLRP3) inflammasome, IGF-1, and VEGF pathways, among others. Among these, four key axes stand out as primary regulators, including the activation of Wnt/β-catenin and PI3K-Akt to promote anagen entry and maintain dermal papilla function, and the inhibition of JAK-STAT and TGF-β to suppress perifollicular inflammation, restrict catagen progression, and help restore HFIP (Fig. 3). Other pathways serve as modulators that enhance these central effects by improving cell survival and redox homeostasis.

Terpenoids chiefly restore hair-cycle dynamics by stabilizing Wnt/β-catenin and activating PI3K-Akt, which drives dermal papilla activity and anagen entry, while some members also temper JAK-STAT or NF-κB. Flavonoids focus on immune rebalancing through suppression of IFN-γ/JAK-STAT and NF-κB, and at the same time support growth and survival pathways such as Akt, ERK, β-catenin, IGF-1 and VEGF. Non-flavonoid polyphenols provide anti-inflammatory and antioxidant support, modulate key stress or cytoskeletal nodes like PAK1 and ERK/p38, and often enhance angiogenesis and follicular resilience. Glycosides and polysaccharides, fatty-acid and quinone derivatives, and alkaloids further integrate immune control with microenvironmental repair by downshifting JAK-STAT or NF-κB, amplifying Wnt or Shh, and improving VEGF-driven perfusion. Collectively, these class-specific actions converge on a unified therapeutic logic that restores HF and anagen readiness while strengthening vascular and redox support, offering a clear basis for rational combination strategies in AA.

Beyond immune and hair cycle modulation, select natural compounds may exert complementary benefits. SalB and GKB upregulate VEGF, potentially improving perifollicular blood flow and nutrient delivery to HFs [53,98]. *Terminalia bellirica* (Gaertn.) Roxb. (TBR) could remodel the follicle microenvironment by reducing oxidative stress and increasing angiogenesis [132]. Certain polyphenols and flavonoids possess metal-chelating properties, enabling them to bind ions like zinc, iron, and copper. This interaction improves compound stability, solubility, and local retention while reducing toxicity. Curcuminoids from turmeric selectively chelate Fe^3+^/Fe^2+^ via β-diketone groups [133]. Similarly, Qc exhibits dose-dependent Fe^2+^ binding, contributing to its antioxidant activity [134]. Importantly, polyphenol-metal complexes, such as epigallocatechin gallate-iron (EGCG-Fe), can self-assemble into nanoparticles, which further enhance cellular uptake and amplify anti-inflammatory effects within biological microenvironments [135]. These interactions highlight the potential of polyphenol-metal chelation in optimizing therapeutic efficacy and delivery. Clinically, botanical-minoxidil combinations resolve complex cases, as evidenced by rapid hair regrowth in a 6-year-old with hidrotic ectodermal dysplasia [136]. Combining 5% *Curcuma aeruginosa* extract with 5% minoxidil significantly improved hair growth and reduced shedding [137]. These findings underscore plant metabolites’ roles as potentiators allowing minoxidil dose reduction, offering multi-mechanistic, well-tolerated options for AA.

Although natural products have demonstrated potential in promoting hair growth and modulating immune responses in cellular and animal models, translating these findings into clinical applications still faces numerous challenges. Currently, most available evidence arises from cell and animal studies rather than well-controlled clinical trials, and existing human studies are often small, short-term, and methodologically inconsistent. Within AA-specific studies, topical 20% azelaic acid performed comparably to 0.5% dithranol over 12 weeks [103], and combinations such as curcumin with piperine and capsaicin have shown signals in small clinical cohorts [97]. Additional human data from hair-loss cohorts (e.g., LIE; *Curcuma aeruginosa* plus 5% minoxidil) are supportive but not AA-specific [123,137]. The lack of standardized preparations across different studies makes it difficult to compare results, due to variations in dosage, purity, and administration methods. Many studies remain preclinical, and researchers note that fundamental differences between mouse and human HFs can confound translation, as robust results in murine models often fail to predict human outcomes [138].

Most limitations extend beyond small and short trials. AA is heterogeneous across phenotype, inflammatory endotype, and relapse, yet most studies do not stratify or assess durability. Product variability from plant source, extraction, and purity, together with incomplete standardization of fingerprints, multi-marker content, stability and impurities, undermines reproducibility [139]. Bioavailability and follicular penetration remain uncertain, with limited pharmacokinetic and pharmacodynamic data. For instance, curcumin and RE exhibit extremely low systemic bioavailability due to rapid metabolism and poor absorption, while ginsenosides and EGCG are similarly constrained by limited intestinal uptake and extensive first-pass metabolism [[140], [141], [142], [143]]. These pharmacokinetic barriers likely contribute to the weak or inconsistent efficacy observed in clinical translation. Furthermore, the clinical translation of natural extracts is hindered by complex compositions, batch variability, and undefined safety profiles, including long-term tolerability and interactions with immunosuppressants or JAKi [144]. These issues, combined with scarce clinical data, often limit natural products to promising concepts without robust validation. Future studies should therefore prioritize the execution of rigorous randomized controlled trials, the development of advanced formulations like nanocarriers to improve bioavailability, and thorough dose optimization to overcome the translational gap between bench and bedside.

By contrast, approved oral JAKi show reproducible, durable SALT-based responses in large randomized trials and therefore set the current benchmark for efficacy and safety monitoring. To move natural products toward clinical use in AA, it is essential to standardize materials using fingerprint chromatograms, multi-marker quantification, and stability and impurity control to ensure batch reproducibility [145,146]. SALT endpoints and durability of regrowth should be aligned with mechanism-linked biomarkers of HFIP restoration and JAK-STAT and Wnt/β-catenin engagement, with functional validation in relevant cell types. Pharmacokinetics and pharmacodynamics should be integrated by quantifying follicular and tissue deposition and modeling the exposure response, and follicle-targeted delivery should be prioritized to overcome bioavailability limits [147]. Run adequately powered, pragmatic trials of at least six to twelve months should be conducted to evaluate add-on or dose-sparing combinations against JAKi or minoxidil, with stratification by severity and inflammatory phenotype. Because formulation and delivery are often limited, bioavailability enhancers and carrier or device platforms such as nanocarriers, liposomes, and microneedles are used to improve follicular deposition, positioning natural products as adjuncts, maintenance options, or steroid and JAKi sparing strategies pending stronger evidence [[147], [148], [149]].

## 6 Conclusion

AA, a prevalent autoimmune dermatological disorder, exhibits a complex pathogenesis involving multiple factors, including dysregulated immune responses, genetic predisposition, neuropsychiatric influences, and potential viral triggers.

In recent years, our understanding of a clearer picture of the mechanisms underlying the breakdown of IP and the role played by specific inflammatory cytokines has significantly deepened. In addressing AA, a diverse array of compounds and medications have exhibited varied efficacies and promising therapeutic advantages. Various treatments, including JAKi, oral minoxidil, and microneedling, have been developed, but more research is needed to understand the interplay of genetics, environment, and therapy response.

The diverse biological activities demonstrated by natural compounds, encompassing their anti-inflammatory, antioxidant, and immunomodulatory capabilities, underscore their potential as therapeutic agents for AA. While traditional therapeutic methods and biological therapies continue to be the cornerstone of AA treatment, their constraints related to contraindications and adverse effects underscore the pressing need for alternative therapeutic options. Natural compounds, terpenoids (e.g., ginsenoside, CE, and PNS), flavonoids (e.g., genistein, and isoflavones), polyphenols (e.g., curcumin, and RE), and other phytochemicals, which have demonstrated potential in promoting hair regrowth and managing AA, offer promising avenues as adjunctive or alternative treatments for AA due to their intricate and multifaceted mechanisms of action.

However, a primary hurdle to advancing natural-compound therapies for AA is the incomplete identification of their *in vivo* molecular targets and mechanisms. Realizing this potential will require rigorous translational work, including the elucidation of *in vivo* targets and pathways of action, standardized manufacturing and quality control of botanical preparations, and pharmacokinetic and pharmacodynamic characterization to define exposure-response and safety margins. This foundational effort must be coupled with adequately powered, well-controlled randomized trials to establish efficacy and delineate risk profiles. Mechanism-guided designs that incorporate biomarkers of immune privilege integrity and cytokine signaling may further enable rational patient stratification and evidence-based combinations with existing treatments, thereby expanding therapeutic options and improving long-term disease management.

## CRediT authorship contribution statement

**Baoli He:** Writing – original draft. **Yujia Weng:** Writing – review & editing, Methodology, Investigation. **Peihua Luo:** Conceptualization. **Zhifei Xu:** Methodology. **Hao Yan:** Methodology. **Bo Yang:** Methodology. **Qiaojun He:** Supervision, Project administration. **Jiabin Lu:** Writing – review & editing, Project administration, Funding acquisition. **Xiaochun Yang:** Supervision, Project administration, Funding acquisition.

## Declaration of competing interest

The authors declare that they have no known competing financial interests or personal relationships that could have appeared to influence the work reported in this paper.
